# Screening of microalgae for integral biogas slurry nutrient removal and biogas upgrading by different microalgae cultivation technology

**DOI:** 10.1038/s41598-017-05841-9

**Published:** 2017-07-14

**Authors:** Xue Wang, Keting Bao, Weixing Cao, Yongjun Zhao, Chang Wei Hu

**Affiliations:** 1 Shanghai Public Green Space Construction Affairs Center, Shanghai, 201100 China; 2 College of Biological Chemical Science and Engineering, Jiaxing University, Jiaxing, 314001 P.R. China

## Abstract

The microalgae-based technology has been developed to reduce biogas slurry nutrients and upgrade biogas simultaneously. In this work, five microalgal strains named *Chlorella vulgaris*, *Scenedesmus obliquus*, *Selenastrum capricornutum, Nitzschia palea*, and *Anabaena spiroides* under mono- and co-cultivation were used for biogas upgrading. Optimum biogas slurry nutrient reduction could be achieved by co-cultivating microalgae (*Chlorella vulgaris*, *Scenedesmus obliquus*, and *Nitzschia palea*) with fungi using the pelletization technology. In addition, the effects of different ratio of mixed LED light wavelengths applying mixed light-emitting diode during algae strains and fungi co-cultivation on CO_2_ and biogas slurry nutrient removal efficiency were also investigated. The results showed that the COD (chemical oxygen demand), TN (total nitrogen), and TP (total phosphorus) removal efficiency were 85.82 ± 5.37%, 83.31 ± 4.72%, and 84.26 ± 5.58%, respectively at red: blue = 5:5 under the co-cultivation of *S. obliquus* and fungi. In terms of biogas upgrading, CH_4_ contents were higher than 90% (v/v) for all strains, except the co-cultivation with *S. obliquus* and fungi at red: blue = 3:7. The results indicated that co-cultivation of microalgae with fungi under mixed light wavelengths treatments was most successful in nutrient removal from wastewater and biogas upgrading.

## Introduction

Increasing energy demands and declining supplies of fossil energy resources have attracted significant attention worldwide^1^. In many places, biogas, as an environmentally friendly renewable energy, is a promising substitute for fossil fuel^2–4^. Biogas produced from landfill and anaerobic digestion processes mainly consists of CH_4_ (50–75% by volume) and CO_2_ (25–50% by volume)^5^. Crude biogas also contains trace amounts of other gases, such as H_2_S, NH_3_, N_2_, O_2_, CO, and H_2_O^6^. Some of these impurities represent health risks for humans and result in increased gas emissions^7^. A number of applications, such as grid injection and vehicle use, require the removal of the high proportions of CO_2_ in biogas to produce a gas with characteristics similar to natural gas^8^. In order to increase the calorific value and decrease the relative density of biogas, it is important to upgrade raw biogas by removing CO_2_ to reach a higher fuel standard^9^.

Biogas is currently experiencing a rapid development, and biogas upgrading is going to be an increasing concern in the bioenergy industry of the future. Biogas upgrading is facing the challenges of energy consumption, operating costs, and environmental risks such as eutrophication^4^. Biogas slurry is a vital byproduct of biogas development, and its processing remains a significant challenge^10^.

The use of microalgae plays a vital role in reducing biogas slurry nutrients and in upgrading biogas, representing a promising technology. Simultaneously, biogas slurry can supply high concentrations of nutrients such as carbon, nitrogen, and phosphorus to produce high microalgae biomass for the production of biochemicals and biofuels^11^. Bhatnagar *et al*.^12^, and Yan *et al*.^13^ suggested that CO_2_ in biogas and nutrients in biogas slurry could be directly removed by microalgae. Both inorganic carbons derived from biogas or organic carbon from biogas slurry could be used to improve microalgae production. Microalgae have extremely high photosynthetic efficiency and carbon biofixation rates, thereby removing CO_2_ and upgrading biogas; they also show a synergistic effect between biogas CO_2_ assimilation and nutrient removal from biogas.

To harvest microalgae, an integrated approach of co-cultivating microalgae with fungi was used in one study; during flocculation, the dispersed algae cells could aggregate and form larger particles^14^. To effectively harvest algae biomass and to establish this symbiosis, it is necessary to develop an effective fungal-algal symbiosis system to assist bioflocculation and to support the removal of wastewater nutrients through immobilized cells^15^. Wrede *et al*.^16^ and Muradov *et al*.^17^ investigated the co-cultivation of microalgae with fungi to remove nutrients from wastewater and generate biomass. Their results indicated that this technique has an exceptional ability to purify wastewater and generate biomass; such obtained biomass represents a renewable and sustainable feedstock for biofuel production. Manheim *et al*.^18^ and Van Den Hende *et al*.^19^ studied the co-cultivation of microalgae with activated sludge to remove nutrients from wastewater, to decrease gas discharge levels, and to produce biomass; the large potential for the application of microalgal bacterial flocs for flue gas sparged sewage treatment. Many factors influence the efficiency of biogas purification and biogas slurry nutrient removal in a photobioreactor (PBR). Combining these two processes is a practical technique since they could promote each other^13^. In a previous study, the effects of mixed light-emitting diode (LED) wavelengths on microalgae growth rates, biogas slurry nutrient removal, and biogas CO_2_ removal were larger than those of monochromatic light wavelengths. A LED is an ideal tool to emit radiation of specific wavelengths to study the influence of light quality on microalgae cultures^20^. Chen *et al*.^21^ and Das *et al*.^22^ stated that red light and blue light were the most important light qualities for microalgal growth. A mix of these two lights could improve growth rates and lipid accumulation of microalgae by enhancing the effects of photosystems I and II.

So far, only a limited number of studies have assessed the combination of three different treatments, microalgae mono-cultivation, microalgae co-cultivation with fungi, and microalgae co-cultivation with activated sludge, in biogas upgrade and biogas slurry nutrients removal. The present study therefore focuses on microalgal strain growth, biogas upgrade, and biogas slurry nutrient reduction using five selected microalgae strains in three cultivation treatments. These five strains used in this work were selected on account of their high growth rates (in the range of 0.327–0.451 d^−1^) and strong nutrient removal ability (more than 60% removal of COD) in wastewater on the basis of our previous research^11^. In the first step, we select the optimal microalgal strains in terms of growth rate and mean daily productivity and then choose the optimum cultivation technology. Subsequently, we evaluate CO_2_ removal and slurry nutrient reduction under different mixed LED light wavelengths, red light and blue light treatments, based on the studied biogas upgrading and biogas slurry purification methods. Based on the results, we then select the optimal strain under the appropriate ratio of red light and blue light for biogas upgrading and biogas slurry nutrient removal. The optimal parameters are determined by analyzing growth rates and mean daily productivity as well as nutrient removal efficiency and biogas purification.

## Results and Discussion

### Growth of the five selected strains under different treatments

Table 1 shows the experimental data for the five microalgal strains (*C. vulgaris*, *S. obliquus*, *S. capricornutum*, *N. palea*, and *A. spiroides*) in terms of growth rates and mean daily productivity under cultures 1, cultures 2, and cultures 3. In general, growth rates were lower under cultures 1 compared to cultures 2 and cultures 3. Cultures 2 has higher growth rates, which was in agreement with the findings of Zhou *et al*.^15^ and Wrede D.^16^, who observed that cultures 2 is highly effective in terms of bioflocculation of microalgal cells and did not require additional chemicals and low energy inputs. Cultures 3 resulted in higher growth rates than cultures 1, but lower than cultures 2. This is because filamentous fungi and activated sludge can potentially entrap or immobilize the microalgae by forming bioflocculation due to their unique filamentous properties^23, 24^. Similar results have been reported by Manheim *et al*.^18^ and Oh *et al*.^25^, who found that bioflocculation by bacteria and their exudates could improve the microalgae settling. Among the five studied microalgal strains in cultures 1, *C. vulgaris* and *S. obliquus* had the relatively high growth rates in all microalgae, respectively, while *A. spiroides* showed the lowest rate. Similarly, mean daily productivity of *C. vulgaris* and *S. obliquus* were higher than *A. spiroides*. *S. obliquus* and *C. vulgaris* had the highest growth rates in cultures 2 and 3, respectively, and *A. spiroides* showed the lowest. Lee *et al*.^26^ increased the tolerance of microalgae to high levels of CO_2_ and nutrients by enhancing the initial microalgal cells density. Microalgal growth could be controlled by microalgae cell density, CO_2_ levels in the biogas, and nutrients in the biogas slurry. Biomass productivity can therefore be a vital parameter to evaluate the CO_2_ and organic carbon removal potential of systems. As seen in Table 1, the cultures 2 and cultures 3 had higher microalgae biomass values than the cultures 1, resulting in higher growth rates. In all three treatments, *C. vulgaris* and *S. obliquus* had the highest mean daily productivity values, while *A. spiroides* had the lowest values. These results suggest that co-cultivation significantly can promote microalgal growth. The fungi improve the microalgal growth mainly on account of the characteristic of fungal cell self-pelletization, which are usually applied for pollutants removal from wastewater^27, 28^. The fungal cell self-pelletization capacity is connected with amphipathic and hydrophobic protein on the hyphal surface, which are adhered to the hyphae^29^. Although the detailed mechanisms of fungal-algal interactions were still unclear, the consensus is the interaction between oppositely charged surfaces may accelerate microalgae absorption by fungal cell wall^16, 17, 27^. Besides, microscopic analysis of algal-fungal pellets demonstrated algal cells was twined by fungal filaments and stick to them^16, 17^.

**Table 1:** Growth rates and mean daily productivity of five microalgae strains under different microalgae cultivation technologies.

Cultivation technology	Mono-cultivation of microalgae	Co-cultivation of microalgae with fungi	Co-cultivation of microalgae with activated sludge
Microalgae strain		Growth rate d^−1^	
*Chlorella vulgaris*	0.361 ± 0.04	0.389 ± 0.03	0.395 ± 0.04
*Scenedesmusobliquus*	0.387 ± 0.02	0.407 ± 0.02	0.383 ± 0.03
*Selenastrum capricornutum*	0.325 ± 0.02	0.368 ± 0.05	0.371 ± 0.02
*Nitzschia palea*	0.346 ± 0.01	0.352 ± 0.03	0.364 ± 0.03
*Anabaena spiroides*	0.301 ± 0.03	0.334 ± 0.04	0.327 ± 0.02
		Mean daily productivity (gL^−1^ d^−1^)	
*Chlorella vulgaris*	0.147 ± 0.023	0.295 ± 0.031	0.179 ± 0.016
*Scenedesmusobliquus*	0.156 ± 0.018	0.321 ± 0.014	0.193 ± 0.010
*Selenastrum capricornutum*	0.113 ± 0.010	0.274 ± 0.011	0.158 ± 0.017
*Nitzschia palea*	0.136 ± 0.019	0.233 ± 0.016	0.141 ± 0.014
*Anabaena spiroides*	0.097 ± 0.012	0.208 ± 0.013	0.116 ± 0.009

### Nutrient removal efficiencies of the different treatments

Nutrient removal from the biogas slurry varied between the three treatments. Table 2 shows the mean COD, TN and TP removal efficiency over 10 days. The combinations *Gl/C. vulgaris* and *Gl/S. obliquus* were most effective in COD removal (*P* < 0.05), while the lowest removal efficiencies were observed for *S. capricornutum* and *A spiroides*. In cultures 2 and cultures 3, COD reduction efficiency of *S. obliquus* reached 75.67 ± 5.78 and 71.76 ± 5.71%, respectively. The five microalgal strains can survive depending on the supply of organic substrates from biogas slurry, and sustain both heterotrophic and autotrophic growth with CO_2_ as the sole carbon source^30^. The carbon content in microalgal biomass exceeds 50%, which demonstrates the importance of carbon assimilation into biomass on microalgal cells formation^30, 31^. Based on our results, the strains *C. vulgaris, S. obliquus*, and *N. palea* can be effectively used in the removal of COD from biogas slurry. This indicates that the appropriate selection of microalgae strains in cultures 2 were effective strategies to enhance COD removal efficiency.

**Table 2:** COD, TN, and P removal efficiencies and CH_4_ concentration in the upgraded biogas during the operational period evaluated.

Three microalgae strains/The mixed LED light wavelength treatments	COD Removal (%)	TN Removal (%)	TP Removal (%)	Concentration of CH_4_ (%, v/v)
Mono-cultivation of microalgae (cultures 1)				
*Chlorella vulgaris*	68.93^a^ ± 4.28	65.29^b^ ± 5.34	64.39^a^ ± 4.76	83.69^a^ ± 5.31
*Scenedesmus obliquus*	70.27^a^ ± 5.03	69.37^a^ ± 4.83	62.55^a^ ± 5.08	85.03^a^ ± 3.28
*Selenastrum capricornutum*	61.04^b^ ± 3.97	59.74^c^ ± 5.27	57.33^b^ ± 5.15	80.56^b^ ± 4.74
*Nitzschia palea*	67.89^a^ ± 5.31	67.86^ab^ ± 4.96	61.29^a^ ± 4.67	84.36^a^ ± 4.57
*Anabaena spiroides*	63.75^b^ ± 4.85	61.38^c^ ± 5.39	56.11^b^ ± 4.03	79.35^b^ ± 3.73
Co-cultivation of microalgae with fungi (cultures 2)				
*Chlorella vulgaris*	73.85^ab^ ± 5.11	71.09^ab^ ± 6.34	70.47^a^ ± 5.62	89.78^a^ ± 3.09
*Scenedesmus obliquus*	75.67^a^ ± 5.78	74.57^a^ ± 5.87	68.39 ^ab^ ± 4.39	90.35^a^ ± 3.77
*Selenastrum capricornutum*	67.59^bc^ ± 4.36	64.18^c^ ± 4.39	59.88^c^ ± 3.97	85.17^b^ ± 2.78
*Nitzschia palea*	70.36^b^ ± 6.09	69.66^b^ ± 5.21	66.26^b^ ± 5.79	88.39^a^ ± 3.25
*Anabaena spiroides*	65.18^c^ ± 4.65	63.37^c^ ± 4.98	61.93^c^ ± 4.31	84.39^b^ ± 3.89
Co-cultivation of microalgae with activated sludge (cultures 3)				
*Chlorella vulgaris*	70.82^ab^ ± 4.73	75.26^ab^ ± 6.32	68.73^a^ ± 5.57	87.37^ab^ ± 4.83
*Scenedesmus obliquus*	71.76^a^ ± 5.71	76.44^a^ ± 5.93	67.24^a^ ± 4.39	90.79^a^ ± 3.11
*Selenastrum capricornutum*	67.37^b^ ± 5.06	70.69^c^ ± 5.38	62.36^b^ ± 5.32	88.68^ab^ ± 3.27
*Nitzschia palea*	72.32^a^ ± 5.47	73.43^bc^ ± 4.65	65.67^ab^ ± 4.08	91.06^a^ ± 2.88
*Anabaena spiroides*	64.38^c ± ^4.58	69.08^c^ ± 5.17	62.44^b^ ± 5.43	83.83^c^ ± 3.31

Note: Values with different superscript letters for the same microalgae cultivation technology indicate a significant difference at *P* < 0.05 according to the Duncan’s multiple range tests.

Among the five strains, *S. obliquus* achieved a significantly higher (*P* < 0.05) TN removal efficiency of 74.57 ± 5.87 and 76.44 ± 5.93% in cultures 2 and cultures 3, respectively, while *S. capricornutum* and *A. spiroides* showed the lowest TN removal efficiencies in the three treatments. The TN is the sum concentration of organic, nitrate, and ammonia nitrogen, while the nitrogen supporting microalgal growth in biogas slurry is ammonia nitrogen^32^. Microalgal reproduction requires sufficient ammonia nitrogen to build nucleic acids and proteins^33^. The combinations *Gl/C. vulgaris*, *Gl/S. obliquus*, and *Gl/N. palea* did not significantly vary with respect to TN removal efficiency (*P* > 0.05). However, their efficiencies were significantly higher (*P* < 0.05) than those of *Gl/S. capricornutum* and *Gl/A. spiroides*. The TN removal efficiencies of *S. capricornutum* and *A. spiroides* were significantly lower (*P* < 0.05) than those of the other microalgal species in cultures 1 and cultures 3 treatments. Tan *et al*.^32^ evaluated the diluted biogas slurry TN removal and *C. vulgaris* showed the highest of 65.87 ± 4.71, which was close to our results. However, when *C. vulgaris* was cultivated with fungus or activated sludge, the TN removal efficiencies reached more than 71% and 75%, which revealed the superiority of cocultivation technology. It is claimed that microalgal-bacterial doesn’t produce NH_4_^+^ because the photoautotrophic microalgae assimilate NH_4_^+^ as their most preferred nitrogen source, which is unlike the biological treatment processes based on bacterial only^34^.

Phosphorus plays a pivotal role in algal production as a constituent of phospholipids and adenosine triphosphate. The highest TP removal efficiency can be found in combinations *Gl/C. vulgaris*, which is 17.7% higher than *Gl/S. capricornutum* (the lowest). Sun *et al*.^35^ measured TP removal efficiencies of co-cultivation of *C. vulgaris*, *S. obliquus*, and *N. oleoabundans* with activated sludge with different CO_2_ concentrations, respectively and reported the TP removal efficiency in the range of 57.92 and 74.11%, which are in keeping with our results. As a result, *C. vulgaris* achieved a significantly higher (*P* < 0.05) TP removal efficiency in the selected five strains, and cultures 2 showed a significantly higher (*P* < 0.05) TP removal efficiency among the three cultivation treatments. The high TP removals of cocultivation of fungi with microalgae (culture 2) is associated with carbohydrate content in microalgae, the high COD reduction accelerate the TP migration^36^. It will result in higher microalgal-bacterial activity and higher TP removals mainly on account of the low dissolved oxygen in the biogas slurry, thus a higher CO_2_ are available for photosynthesis^37^.

### Biogas upgrading

The CH_4_ concentration represented the biogas upgrade level, the higher CH_4_ concentration, the higher biogas upgrade level. According to Tables 2 and 3, in cultures 2 and cultures 3, CO_2_ could be significantly removed from biogas (*P* < 0.05). The *P*-values for the CH_4_ parameter in Table 3 show that the interaction of the three different cultivation treatments and microalgae strains significantly influenced CH_4_ concentrations (*P* < 0.05), while there were no significant effect of microalgae strains on CH_4_ concentration (*P* > 0.05). In the present study, the combination *Gl/S. obliquus* had the highest CH_4_ concentration (90.35 ± 3.77%), which was significantly higher than other treatments (*P* < 0.05). The high CO_2_ reduction using *C. vulgaris* lead to the CH_4_ enrichment because of the photosynthesis of microalgae. Therefore, cultures 2 seemed to be the optimal treatment for biogas upgrading. Moreover, high mean daily productivity (g L^−1^ d^−1^) of microalgae and nutrient removal efficiency showed that *C. vulgaris* can effectively be used in biogas purification, followed by *S. obliquus*. In cultures 2 and cultures 3, most of the fungal-microalgal pellets could efficiently remove CO_2_ from biogas and reach the efficient combustion standard when the CH_4_ concentration > 90% (v/v)^8^, with the exception of using *A. spiroides* and *S. capricornutum*. The microalgal strains can change their metabolic pathway based on the amounts of organic substrates, such as organic acids and glucose, suggesting that they can grow by heterotrophic way in addition to common autotrophic growth and use CO_2_ as the sole carbon source^30^.

**Table 3:** P-values of factors and combined effects of factors for each parameter under different treatment technology based on analysis of variance.

Factors	COD Removal (%)	TN Removal (%)	TP Removal (%)	Concentration of CH_4_ (%, v/v)
Microalgae species	0.058	0.044*	0.037*	0.058
Treatment technology	0.026*	0.039*	0.023*	0.015*
Microalgae species × treatment technology	<0.001**	<0.001**	<0.001**	0.029*

Note: Microalgae species: *C. vulgaris* FACHB-31, *S. obliquus* FACHB-416, *S. capricornutum* FACHB-271, *N. palea* FACHB-211, and *A. spiroides* FACHB-498; Treatment technology: Mono-cultivation of microalgae (cultures 1), Co-cultivation of microalgae with fungi (cultures 2), Co-cultivation of microalgae with activated sludge (cultures 3). *P < 0.05, **P < 0.01.

### Microalgae-fungal strain growth at various mixed LED light wavelength treatments

Table 4 presents the daily productivity of *Gl/C. vulgaris*, *Gl/S. obliquus*, and *Gl/N. palea* over a period of 10 days under various mixed LED light wavelength treatments. *Gl/S. obliquus* has the maximal mean daily productivity of 0.353 ± 0.019 under the mixed LED light wavelength ratio of red: blue = 5:5. In all treatments, the mean daily productivity of mixed LED light wavelength ratio red: blue = 5:5 was the highest. The microalgae grown better under mixed LED light wavelength treatments than the white LED light wavelength when compared with the results showed in Table 1. The results are in agreement with the findings of Yan *et al*.^38^, who compared the effects of three mixed and white LED light wavelength treatments on growth of *Chlorella sp*. When fungi were cultured with microalgae in biogas slurry, the fungal were pelletized through bioflocculation and non-bioflocculation. The cocultivation way resulted in the large specific surface area of algae-fungi symbionts and the nutrient intake capacity^39^.

**Table 4:** Mean values ± SD of daily productivity, as well as the removal efficiency of biogas slurry nutrients and CO_2_ at various light wavelength treatments under cultures 2 for the three selected microalgae strains.

Microalgae strains/The mixed LED light wavelength treatments	Daily productivity (gL^−1^ d^−1^)	COD Removal (%)	TN Removal (%)	TP Removal (%)	CO_2_ Removal (%)
*Chlorella vulgaris*					
Red (7): Blue (3)	0.318 ± 0.013	81.39^ab^ ± 5.14	75.02^b^ ± 4.39	79.28^ab^ ± 4.48	85.15^a^ ± 4.68
Red (5): Blue (5)	0.336 ± 0.025	84.46^a^ ± 4.95	79.06^a^ ± 5.57	81.33^a^ ± 5.83	78.51^b^ ± 3.61
Red (3): Blue (7)	0.302 ± 0.011	77.44^b^ ± 5.81	72.15^b^ ± 5.24	76.21^b^ ± 6.37	79.89^b^ ± 5.77
*Scenedesmus obliquus*					
Red (7): Blue (3)	0.329 ± 0.022	83.16^ab^ ± 6.83	81.09^ab^ ± 6.63	82.07^b^ ± 4.87	82.18^a^ ± 3.86
Red (5): Blue (5)	0.353 ± 0.019	85.82^a^ ± 5.37	83.31^a^ ± 4.72	84.26^a^ ± 5.58	79.92^ab^ ± 5.37
Red (3): Blue (7)	0.315 ± 0.016	80.14^b^ ± 4.68	76.22^b^ ± 5.31	81.08^b^ ± 6.35	75.63^b^ ± 4.54
*Nitzschia palea*					
Red (7): Blue (3)	0.284 ± 0.012	79.47^a^ ± 5.22	75.68^a^ ± 3.98	76.14^a^ ± 6.04	85.59^a^ ± 5.93
Red (5): Blue (5)	0.316 ± 0.010	81.16^a^ ± 3.99	77.96^a^ ± 6.39	78.17^a^ ± 6.57	80.46^b^ ± 4.67
Red (3): Blue (7)	0.271 ± 0.013	75.79^b^ ± 5.74	71.56^b^ ± 6.57	72.26^b^ ± 4.65	78.23^b^ ± 6.35

Note: Values with different superscript letters in the same microalgae strain indicate a significant difference at *P* < 0.05 according to Duncan’s multiple range tests.

### Nutrient removal efficiencies at various mixed LED light wavelength treatments

**Figure 1:**
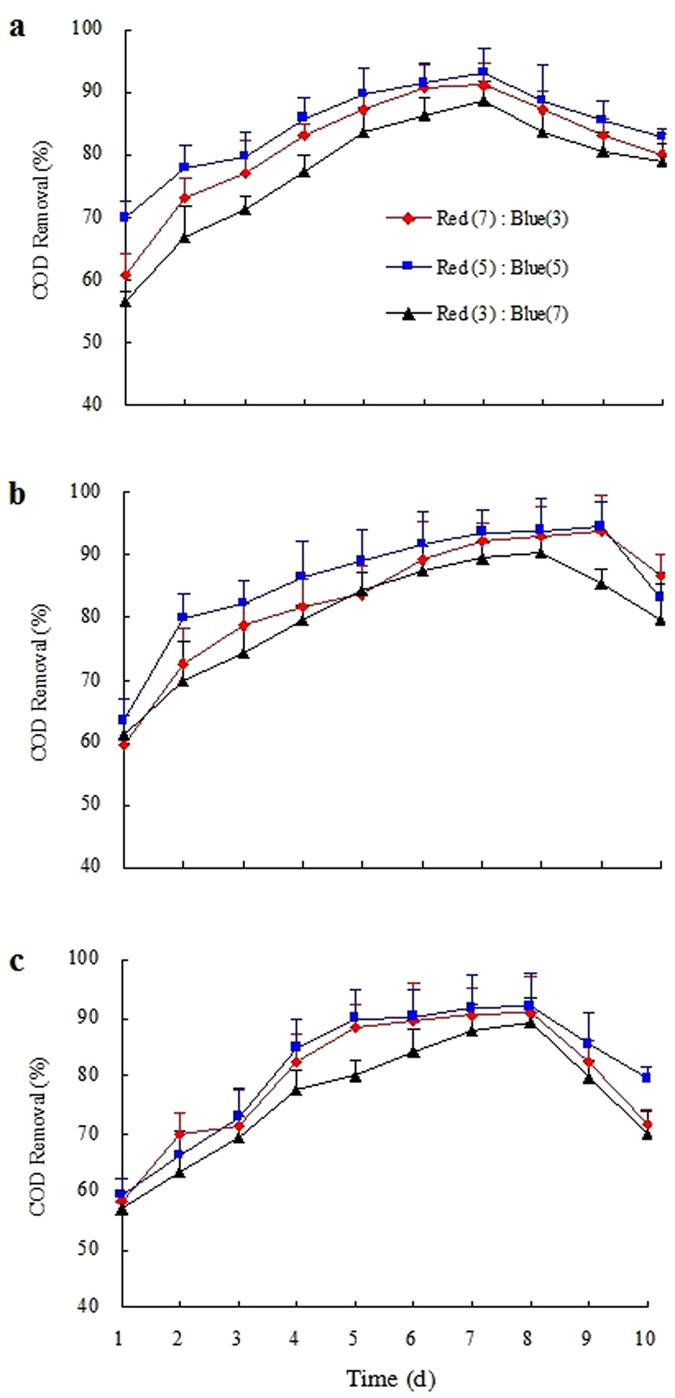
COD removal efficiency over time at cultures 2 under the mixed LED light wavelength treatments for the three selected microalgae species: (**a**) *Chlorella vulgaris*, (**b**) *Scenedesmus obliquus*, and (**c**) *Nitzschia palea*.

COD removal efficiency in various ratios of mixed red and blue LED light wavelengths during a 10-day culture are shown in Fig. 1. The combinations *Gl/C. vulgaris*, *Gl/S. obliquus*, and *Gl/N. palea* showed similar trend of COD removal efficiencies (Fig. 1a–c) under different mixed light wavelength ratio of red: blue. It was increased dramatically in the first six days, and then slightly increased in the following days. The combinations *Gl/C. vulgaris*, *Gl/S. obliquus*, and *Gl/N. palea* showed the highest COD removal efficiency (more than 90%) at the 7^th^, 9^th^, and 8^th^ day, respectively. The removal efficiency finally decreased until the 10^th^ day under the mixed LED light wavelength ratio of red: blue = 5:5. The COD removal efficiency achieved by *Gl/S. obliquus* in red: blue = 5:5 was significantly higher than those of the other treatments (*p* < 0.05). Research has shown that fungi can consume carbohydrates and other nutrients produced during the photosynthesis of microalgae. The fungi will also capture the minerals and other nutrients because of its water-retaining property^40^. Therefore, red: blue = 5:5 was considered as the optimal mixed light wavelength ratio of red: blue for COD removal using *Gl/S. obliquus* treatment (Table 4).

**Figure 2:**
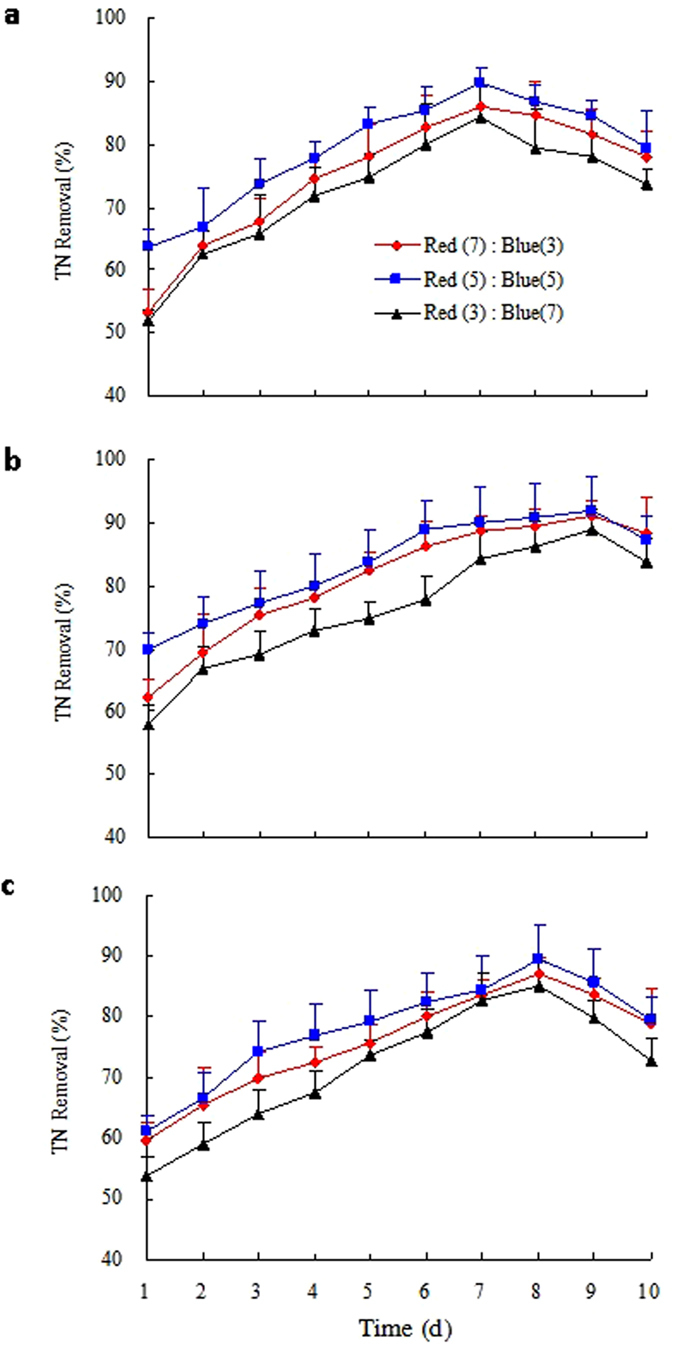
TN removal efficiency over time at cultures 2 under the mixed LED light wavelength treatments for the three selected microalgae species: (**a**) *Chlorella vulgaris*, (**b**) *Scenedesmus obliquus*, and (**c**) *Nitzschia palea*.

**Figure 3:**
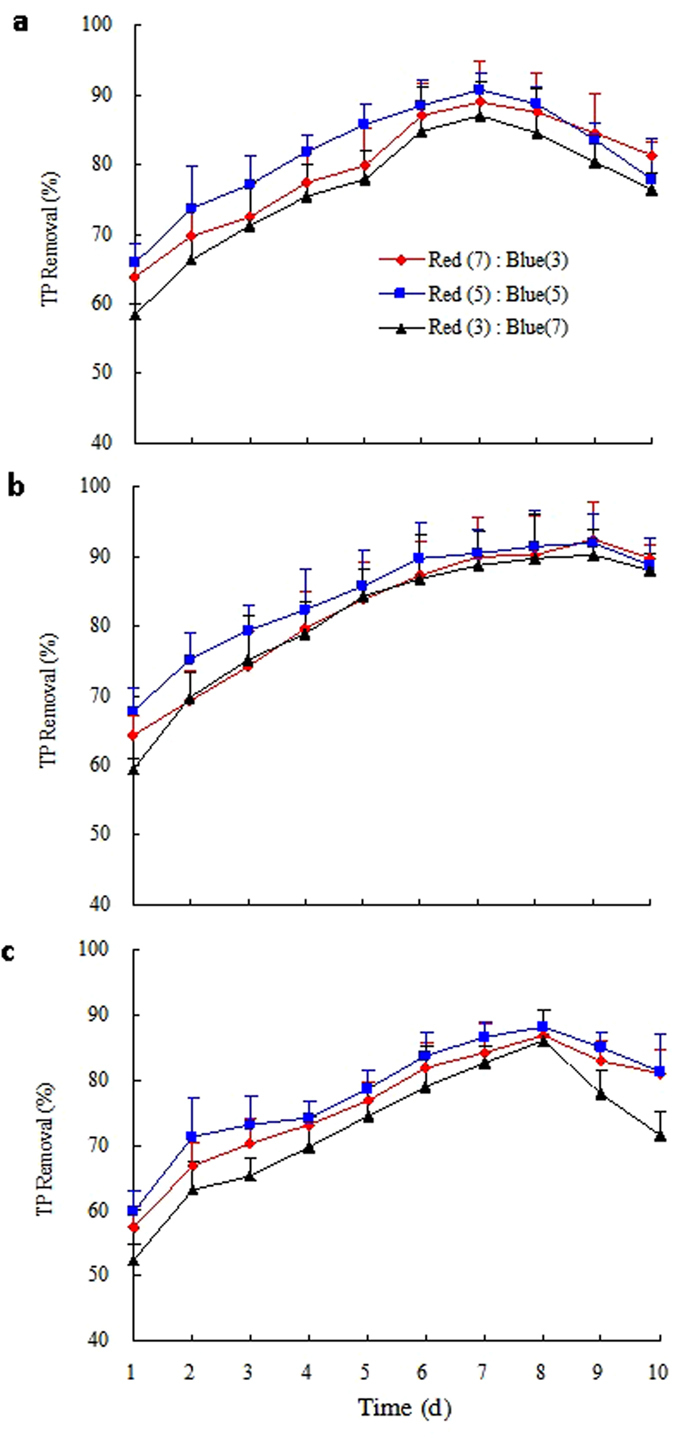
TP removal efficiency over time at cultures 2 under the mixed LED light wavelength treatments for the three selected microalgae species: (**a**) *Chlorella vulgaris*, (**b**) *Scenedesmus obliquus*, and (**c**) *Nitzschia palea*.

Figures 2 and 3 shows the TN and TP removal efficiency in various mixed red and blue LED light wavelengths ratios during a 10-day culture, respectively. The combinations *Gl/C. vulgaris*, *Gl/S. obliquus*, and *Gl/N. palea* pellets of TN and TP removal efficiency under different light qualities (Figs 2 and 3) increased dramatically within 7 and 5 days. *Gl/S. obliquus* has the highest TN removal efficiency on the 9^th^ day, while the combinations *Gl/C. vulgaris* and *Gl/N. palea* achieved relatively high TP removal efficiencies on the 7^th^ and 8^th^ day, respectively. As seen in Table 4, the maximum mean TN removal was also found when using *Gl/S. obliquus* under the wavelength ratio of red: blue = 5:5. To be different, *Gl/S. obliquus* (92.34%) showed the highest TP removal efficiency in the 9^th^ day under red: blue light wavelength ratio of 7:3. The maximal mean TP removal efficiency (84.26%) of *Gl/S. obliquus* was found under the wavelength ratio red: blue of 5:5. These findings revealed that the selected mixed wavelength light for microalgal growth improved the photosynthetic efficiency. The adequate ratio of mixed red and blue light source is good for nitrogen and phosphorus capturing from biogas slurry. It is an efficient alternative way to increase nitrogen and phosphorus removal rates^41^. Predilection of red or blue light in the mix leads to a negative effect on phosphorus removal. As a result, the appropriate mixed LED light wavelength ratio of red: blue for TN removal using *Gl/S. obliquus* is 5:5 when in view of the COD and TN removal efficiency.

### Biogas upgrading at various mixed LED light wavelength treatments

**Figure 4:**
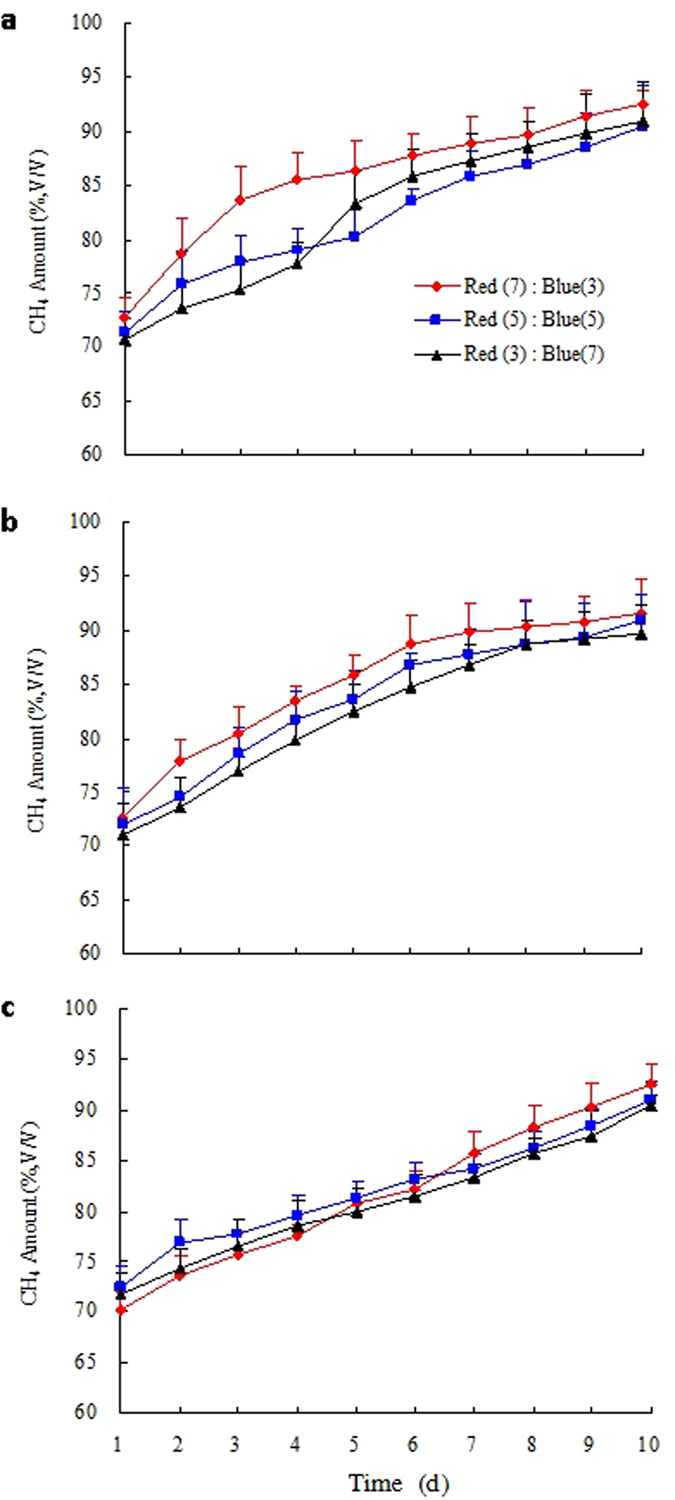
The CH_4_ amount (v/v) of biogas over time at cultures 2 under the mixed LED light wavelength treatments for the three selected microalgae species: (**a**) *Chlorella vulgaris*, (**b**) *Scenedesmus obliquus*, and (**c**) *Nitzschia palea*.

*Gl/C. vulgaris*, *Gl/S. obliquus*, and *Gl/N. palea* were used for CO_2_ removal in a 10-day culture (Fig. 4a–c; Table 4). The CH_4_ content first increased dramatically, and then slightly increased in the following days. However, the CO_2_ removal efficiency of *Gl/N. palea* almost increased throughout the 10 days. All three strains under the three different mixed LED light wavelength treatments resulted in more than 90% of CH_4_ in biogas, which were equivalent to those of natural gas. The CH_4_ amount under mixed LED light wavelength ratio red: blue of 7:3 was higher than those of 5:5 and 3:7, respectively. However, Yan *et al*. found that *Chlorella sp*. increased microalgal growth and achieved the highest CO_2_ removal efficiency under the mixed LED light wavelength ratio red: blue of 5:5^13, 38^. Therefore, red and blue light with the wavelength of 620–750 nm and 476–495 nm are beneficial to the photosynthesis of microalgae, the suitable mix ratio are better for the growth of microalgae than providing a single wavelength^38^. The microalgal reproduction is largely dependent on the utilizable light wavelengths. With the optimal mixed LED light wavelength ratio red: blue of 7:3 cause an efficient CO_2_ removal efficiency. This phenomenon was different from the results of Yan and Zheng mainly because of the different microalgal strains^42^. So, *Gl/N. palea* could achieve the highest CO_2_ removal efficiency under the mixed LED light wavelength ratio red: blue of 7:3.

## Conclusions

Co-cultivation of microalgae with fungi or activated sludge showed higher growth rates and mean daily productivity when compared to mono-cultivation. Among the five strains, *C. vulgaris*, *S. obliquus*, and *N. palea* achieved the nearly high COD, TN, TP, and CO_2_ removal efficiency in cultures 2. Biomass growth was not always directly related to nutrient and CO_2_ removal. Although *S. obliquus* demonstrated the highest growth efficiency in the three treatments, it was not the best for TP and CO_2_ removal efficiencies. Based on our results, the mixed LED light wavelength ratio red: blue of 5:5 for nutrients removal using *Gl/S. obliquus* was recommended as the optimum. Meanwhile, the mixed LED light wavelength ratio red: blue of 7:3 using *Gl/N. palea* yielded optimal results in terms of CO_2_ removal. Thus, the microalgae-based photo-bioreactor used in this study can effectively upgrade biogas and reduce nutrients simultaneously.

## Methods and Materials

### Collection of algal and fungi strains and culturing conditions

We selected five microalgae strains, Chlorella vulgaris (C. vulgaris; FACHB-31), Scenedesmus obliquus (S. obliquus; FACHB-416), Selenastrum capricornutum (S. capricornutum; FACHB-271), Nitzschia palea (N. palea; FACHB-211), and Anabaena spiroides (A. spiroides; FACHB-498). All strains were obtained from the Institute of Hydrobiology at the Chinese Academy of Sciences. The strains C. vulgaris, S. obliquus, and A. spiroides were preserved in standard BG11 medium, while N. palea was preserved in CSI medium and S. capricornutum in SE medium. The culturing conditions were as follows: white LED light with an intensity of 200 μmolm^−2^ s^−1^, 25 ± 0.5 °C, light: dark = 14 h: 10 h, and artificial intermittent shaking four times a day.

A strain of the fungus *Ganoderma lucidum* (*G. lucidum*; 5.765) was obtained from the China General Microbiological Culture Collection Center. After a three-day pre-cultivation, the *G. lucidum* spores were washed from the potato dextrose agar (PDA) medium with sterile water and transferred into 1 L potato dextrose broth (PDB) medium. The spore solution was then cultivated at 28 ± 0.5 °C. To obtain pellets, spore solutions were cultivated at 28 ± 0.5 °C for 72 h in a 1 L synthetic medium (glucose, 10 g·L^−1^; NH_4_NO_3_, 2.0 g·L^−1^; K_2_HPO_4_, 1.0 g·L^−1^; NaH_2_PO_4_·H_2_O, 0.4 g·L^−1^; MgSO_4_·7H_2_O, 0.5 g·L^−1^; and yeast extract, 2.0 g·L^−1^; pH 6.5). Algal cultures were concentrated, washed and resuspended to achieve a final concentration of 103.26 ± 7.39 mg L^−1^ before being added to the collected fungus for pelletization. The fungal-algal mixtures were shaken at 160 rpm for 48 h under constant light (200 μmol·m^−2^ s^−1^) and kept under a light-dark cycle of 14 h: 10 h at 25 ± 0.5 °C.

The five selected algae strains were cultivated in biogas slurry, which was inoculated with 1 L of a 0.75 g total suspended solid (TSS) L^−1^ and 200 mL of a 4.14 g TSS L^−1^ nitrifying-denitrifying activated sludge from a wastewater treatment plant in Shanghai, China. The initial concentration of TSS in the cultivation biogas slurry was about 0.06 g·L^−1^.

### Photobioreactor (PBR)

**Figure 5:**
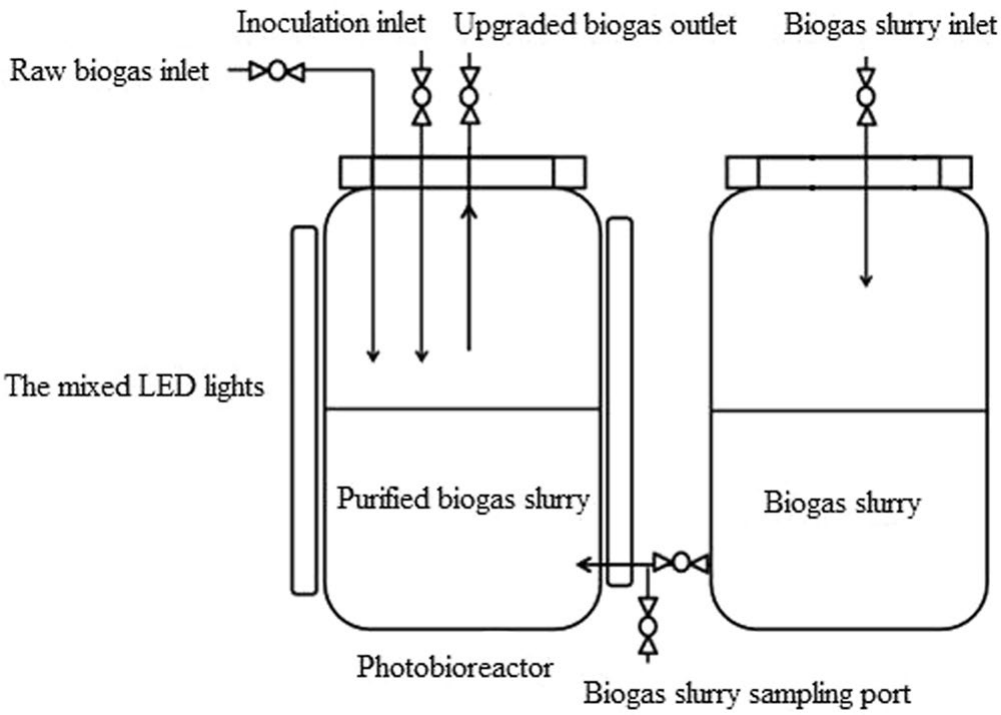
Schematic of the photobioreactor experimental setup.

The PBR was formed by two interconnected and individual 16.8 L glass cylinder blocks (Fig. 5). The two cylinder blocks had a total height of 0.6 m and a diameter of 0.2 m. The PBR were hermetically sealed with rubber stoppers. The biogas slurry was filtered using a glass microfiber filter (GF/C; Whatman, USA) to remove large particles before being sterilized with an ultraviolet sterilizer (SKW-UVU01; SKYUV Water Treatment Co. Ltd, China) for 2 min, and then pumped into the right tank. Biogas slurry (2.8 L) was pumped from the right to the left cylinder block of the PBR. Raw biogas (14 L) was injected into the left reactor. The mono-cultured microalgae, fungal-microalgal pellets and microalgae assisted with activated sludge were added to the left reactor before being illuminated by LED lights at 200 μmol m^−2^ s^−1^ arranging in a circular configuration (20 W, 110 V). The carbon, nitrogen and phosphorus in biogas slurry will provide the nutrient for microalgae growth. The CO_2_ in biogas and light will provide raw materials for photosynthesis of microalgae. The biogas in PBR was sampled from the upside of PBR for gas component analysis every 24 h after the PBR started. The biogas slurry was sampled from the downside of the PBR for analysis of COD, TN and TP.

Table 5 shows the characteristics of the biogas slurry and crude biogas for the three treatment technologies in this work. Crude biogas was fed via PBR headspace. We determined the effects of the appropriate technology under different mixed LED light wavelength treatments on the growth rate, mean daily productivity, nutrient removal efficiency, and biogas purification. The culture conditions were similar to algal and fungi strains and culturing conditions. The microalgae were cultured for 10 days.

**Table 5:** The characteristics of biogas slurry and crude biogas.

Parameter	Before pretreatment	After pretreatment
pH	6.89 ± 0.08	6.81 ± 0.05
DIC (mgL^−1^)	1016.26 ± 35.44	999.03 ± 39.81
COD (mgL^−1^)	993.15 ± 49.21	978.35 ± 52.06
TN (mgL^−1^)	347.28 ± 18.19	341.54 ± 12.87
TP (mgL^−1^)	46.77 ± 6.33	39.25 ± 5.14
CH_4_ (%, V/V)	—	67.21 ± 3.72
CO_2_ (%, V/V)	—	29.63 ± 2.19
H_2_O (%, V/V)	—	2.95 ± 0.31
O_2_ (%, V/V)	—	0.21 ± 0.08
H_2_S (%, V/V)	—	<0.005

### Biogas slurry and biogas

Biogas was obtained from a farm biogas plant in JiaYuan Green Meadow. Prior to the experiments, biogas was desulfurized in chemical absorption reactors to reduce H_2_S concentration to value below 100 ppm.

### Experimental procedure

#### Experiment 1

Mono-cultivation of microalgae (cultures 1): Microalgal suspensions (100 mL; 119.72 ± 8.13 mg·L^−1^ dry weight) of *C. vulgaris*, *S. obliquus*, *S. capricornutum*, *N. palea*, and *A. spiroides* were prepared.

Co-cultivation of microalgae with fungi (cultures 2): Microalgal suspensions (100 mL; 115.63 ± 7.39 mg·L^−1^ dry weight) of *C. vulgaris*, *S. obliquus*, *S. capricornutum*, *N. palea*, and *A. spiroides* were prepared, and each suspension was mixed with 5 mL of *G. lucidum* pellet suspension (78.34 ± 6.05 mg·L^−1^ dry weight). The initial density of the microalgae co-cultivated with fungal cells was maintained constant at 117.47 ± 5.98 mg·L^−1^.

Co-cultivation of microalgae with activated sludge (cultures 3): The biogas slurry was inoculated with 1 L of a 1.50 g TSS L^−1^ for each microalgal strain culture and with 200 mL of an 8.39 g TSS L^−1^ nitrifying-denitrifying activated sludge from a wastewater treatment plant in Shanghai, China. Initial TSS concentration in the cultivation broth of the biogas slurry was 121.06 ± 6.36 mg·L^−1^. The amount of bacteria inoculated in the biogas slurry was twice as high as that of the microalgae to ensure a rapid biodegradation start-up.

Initial dry weight of mono-cultivation of microalgae, co-cultivation of microalgae with fungi, and co-cultivation of microalgae with activated sludge before being injected the PBR was about 120 mg·L^−1^. The experiment was conducted 25.0 ± 0.5 °C with the light intensity of 200 μmol m^−2^ s^−1^ and a white light-dark cycle of 14: 10 h (light was provided only from 8:00 am. to 10:00 pm.) for 10 d (240 h).

#### Experiment 2

The optimal ratios of mixed LED light wavelengths for efficient nutrient removal of biogas slurry and biogas purification under the selected cultivation treatment were studied at various light wavelength ratios of red: blue (3:7, 5:5, and 7:3) at constant light intensity of 200 μmol m^−2^ s^−1^ for the left-cylinder block over 10 days. In this experiment, the PBR was also filled with 14 L of raw biogas and 2.8 L of biogas slurry. All treatments were performed at a temperature of 25.0 ± 0.5 °C and a light: dark cycle of 14: 10 h.

The effects of different treatments on biogas upgrading and biogas slurry purification as well as on microalgal growth were evaluated in two steps. The first step was to select three dominant microalgal strains under the irradiation of the same white light. In the second step, the optimization of biogas slurry purification and biogas upgrading technology using the selected three strains in the first step based on different mixed light wavelength control strategy.

### Sampling and analyses

All treatments in all PBRs were sampled daily from the beginning of the inoculation. The dry weight was measured as follows: Culture suspensions of 100 mL were filtered using a glass microfiber filter (GF/C, Whatman, USA). The microalgae were then attached to the filter, which was subsequently dried at 100 °C for 24 h and then cooled to room temperature in a desiccator. Finally, dry weight was calculated from the difference between the filter weights before and after filtration. Concentrations of CH_4_, and CO_2_ (v/v) in the biogas were analyzed by a gas analyzer (GA94, ONUEE Co., Ltd., China)^13^. The culture filtrates were analyzed for COD, TN, and TP concentrations by using standard methods^43^.Biomass productivity (*P*, g L^−1^ d^−1^) was calculated using Eq. (1):

1 $$P=({D}_{i}-{D}_{0})/({t}_{i}-{t}_{0})$$

Specific growth rate (*µ*) was calculated using Eq. (2)^44^:

2 $$\mu =(\mathrm{ln}\,{D}_{i}-\,\mathrm{ln}\,{D}_{0})/{t}_{i}$$

where *D*_i_ is the biomass concentration (g L^−1^) at time *t*_i_ (d) and *D*_0_ is the initial biomass concentration (g L^−1^) at t_0_ (d).Biogas CO_2_ or nutrient removal efficiency (*R*, %) was calculated using Eq. (3):

3 $$R=(1-{C}_{i}/{C}_{0})\times 100$$

where *C*_i_ is the biogas CO_2_ content (%, v/v) or nutrient concentration (g L^−1^) in the filtrates of the cultures at time *t*_i_ and *C*_0_ is the initial biogas CO_2_ content (%, v/v) or nutrient concentration (g L^−1^) at *t*_0_ (day).

### Statistical analyses

One-way analysis of variance (ANOVA) was used to test the statistical difference of the related parameters of the five algae strains using the same light treatment technology. Duncan’s multiple range tests were used to further assess differences among those algae strains that were significant in ANOVA. Two-way ANOVA was used to test for differences between the effects of treatment technology, algae strains, and to detect possible interactions between any two of these factors and their impacts on treatment performance. *P* = 0.05 and *P* = 0.01 were used as thresholds for statistical significance. All analyses were performed using SPSS (V19.0).
